# Immobilization of Magnetic Nanoparticles on Cellulosic Wooden Sawdust for Competitive Nudrin Elimination from Environmental Waters as a Green Strategy: Box–Behnken Design Optimization

**DOI:** 10.3390/ijerph192215397

**Published:** 2022-11-21

**Authors:** Manasik M. Nour, Maha A. Tony, Hossam A. Nabwey

**Affiliations:** 1 Department of Mathematics, College of Science and Humanities in Al-Kharj, Prince Sattam bin Abdulaziz University, Al-Kharj 11942, Saudi Arabia; 2 Basic Engineering Science Department, Faculty of Engineering, Menoufia University, Shebin El-Kom 32511, Egypt; 3 Advanced Materials/Solar Energy and Environmental Sustainability (AMSEES) Laboratory, Faculty of Engineering, Menoufia University, Shebin El-Kom 32511, Egypt

**Keywords:** biowaste, nanoparticles, Fenton’s oxidation, Nudrin, wastewater

## Abstract

The role of engineering in our society is not to just to continue creating chemicals, but sharing the responsibility for environmentally sound appropriate design of substances for a circular economy. Concerning this contemporary strategy, waste wooden sawdust (WSD) as a biobased by-product is augmented with magnetite (Mag) nanoparticles to meet the concept of cyclic application of resources in environmentally relevant photocatalytic reactions. The physical properties of the prepared WSD:Mag material were characterized to emphasis their structure and morphology by using X-ray diffraction (XRD) and transmission electron microscopy (TEM), then the prepared catalyst was applied in augmentation with hydrogen peroxide as a type of photocatalyst in the form of Fenton’s reaction system to oxidize Nudrin pesticide in queues media. Twinned WSD:Mag has been verified to exhibit higher performance than pristine single-phase catalysts. System parameters, i.e., pH, hydrogen peroxide, catalyst dozing, and temperature, were studied to check their effect on the reaction activity. In the present study, further promotion of photocatalytic activity of twinned WSD:Mag was obtained by optimizing the process parameters at the optimal reaction time of 30 min. The optimal results investigated via Box–Behnken design regression model based on response surface mythology (RSM) showed that the photocatalytic activity of the twinned catalyst could reach 94% at pH 2.5 and 386 and 38 m/L of H_2_O_2_ and WSD:Mag, respectively. The regression coefficient and probability obtained from analysis of variance (ANOVA) were used to check the adequacy of the applied model, and were 92% and 0.02, respectively. Additional confirmatory tests were carried out under optimum conditions for verification and agreed with the predicted values. Experimental data analysis revealed that the reaction is well fitted with the second-order reaction model. Thermodynamic parameters highlighted the oxidation reaction is non-spontaneous at high temperature and exothermic in nature and proceeds at a low activation energy barrier (31.46 kJ/mol). Catalyst recyclability was also checked, which confirmed catalyst sustainability and high removal rates (78%) after six cycles of use. This work introduces a new concept to design a promising environmentally benign photocatalyst with high potential for applicability to environmental remediation of agricultural effluents with a view to a circular economy.

## 1. Introduction

In recent years, the new European bioeconomy strategy has focused on endorsing the sustainable use of renewable resources, such as wood-based by-products [1]. The target is to valorize waste into new valuable biobased products. In this regard, the use of waste sawdust material technology is one such bioeconomy strategy. The application of photocatalysis is a suitable candidate to tackle the global environmental and energy challenges. Although various types of photocatalysis have been used for different types of wastewater treatment, real applications are still sparse [2,3,4].

The classical Fenton reaction, one of the photocatalytic systems, is comprised of hydrogen peroxide and Fe^2+^ salts, and Fe^3+^ salts can also be used in the so-called Fenton-like reaction. In acidic aqueous media, both systems, Fenton and Fenton-like reactions, can produce highly reactive hydroxyl (OH) radicals. Hydroxyl radicals are nonselective species that can oxidize different types of pollutants. Such radicals can oxidize organic compounds into atoxic species—CO_2_ and H_2_O [5,6]. Furthermore, a short reaction time is needed for complete oxidation [4]. Although Fenton and Fenton-like processes exhibit high oxidizing efficiencies in aqueous effluent remediation, some drawbacks still exist in real applications, such as iron sludge after treatment [7,8]. Selecting an appropriate, cost-efficient, and sustainable material that possesses unique and attractive catalytic activity has received great attention in wastewater treatment applications. The abundant research published has focused on using fresh iron salt as the source of Fenton’s reaction. However, the source of chemicals is still expensive and unsustainable [9,10,11,12]. For instance, Zhao et al. [5] used iron chloride as a source of Fenton’s oxidation in their system. Thabet et al. [7] treated azo dye in synthetic wastewater effluent using iron particles. Tony and Lin [9] applied iron augmented with hydrogen peroxide as the source of their reaction for eliminating pesticide. It is worth mentioning that the application of iron oxides in heterogeneous-form Fenton-like reaction has several advantages over homogeneous systems [9]. In such heterogeneous methodology, the catalysts are easily recoverable since they can be separated and their activity can also be retained for subsequent treatments [1,10,11,12]. Thus, this could avoid secondary contamination and reduce actual implementation cost. Moreover, from a commercial point of view, valorizing a waste material to be a photocatalyst is gaining scientists’ attention.

The persistent need of out-of-season fruit and vegetables with high-quality yields has forced the creation innovative systems for cultivating in greenhouses in order to assure protected conditions for the crops [13]. Advanced greenhouses have emerged that can recover the surplus water that is not used by vegetation in order to create a semiclosed recycling infrastructure for efficient use of environmental resources [14]. However, to avoid the phytotoxicity that can occur due to the accumulated pesticides after several cycles, a treatment prior to its reuse and recirculation is urgent. Since pesticides are recalcitrant and bioaccumulative, the traditional separation techniques are insufficient to achieve sufficient water quality for recirculation [15,16,17]. Therefore, conventional biological methodologies are not adequate. In addition, physical separation through membranes only transfers the pollutants from one phase to another without mineralizing them. Thus, searching for mineralizing pollutants rather than transferring them to another phase is gaining researchers’ interest. A chemical oxidation system that is able to mineralize such substances is required [18,19]. Fenton’s reaction based on the ultraviolet reaction could be a possible option.

To remedy the problem of waste sludge by-product after Fenton reaction, as well as reduce the cost of the reagents used, this study focused on improvement of the classical Fenton reaction by using a magnetite-based catalyst augmented with treated sawdust as a recoverable composite material. This new photocatalyst was employed for Nudrin pesticide elimination from aqueous streams. There is a lack in the published articles that focus on the application of biowaste material in its magnetized form as a photocatalyst to conduct a Fenton oxidation test. The current work overcomes the demerits of Fenton’s reaction by utilizing waste stream material in a circular economy fashion. As a result, the current study approach is emphasis dual eco-sustainability by using biowaste in treating aqueous effluent waste. Optimization of oxidation system parameters, i.e., WSD:Mag catalyst dose, hydrogen peroxide concentration, effluent pH, Nudrin loading, and temperature, was conducted to maximize the system yield.

## 2. Experimental Investigation

### 2.1. Preparation of Wooden Sawdust–Magnetite Photocatalyst (WSD:Mag)

Firstly, magnetite (Mag) nanoparticles were prepared via coprecipitation from precursors of ferrous sulfate (FeSO_4_·7H_2_O) and ferric sulfate (Fe_2_(SO_4_)_3_), supplied by Qualikems Fine ChemPvt. Ltd., Gujarat, India. The precursors were mixed at stoichiometric ratios with distilled water followed by dropwise addition of NaOH solution till pH 11.0 had been achieved. During the continuous stirring of the solution at 80 °C, a precipitate was obtained. The solution was then successively washed to reach to pH 7.0 and the collected precipitate (Fe_3_O_4_) subjected to oven drying (60 °C) [20].

After collection from a local carpentry facility, wooden sawdust (WSD) was washed repeatedly in distilled water prior to drying at 105 °C in order to isolate the cellulosic fibers. The clean dried powder was then hydrolyzed in HCl at 90 °C for 15 min through stirring. Consequently, the acquired mixture was filtered then washed repeatedly to reach a neutral pH. The obtained cellulosic fibers were dried at 60 °C until a constant weight was achieved, followed by ball milling (300 rpm, 10 h). The resultant powder was bleached with H_2_O_2_ (3%) through stirring for one hour at 90 °C. The resultant mixture was washed repeatedly in distilled water to reach pH 7.0, then dried, and an isolated cellulosic substance was obtained [21].

**Figure 1:**
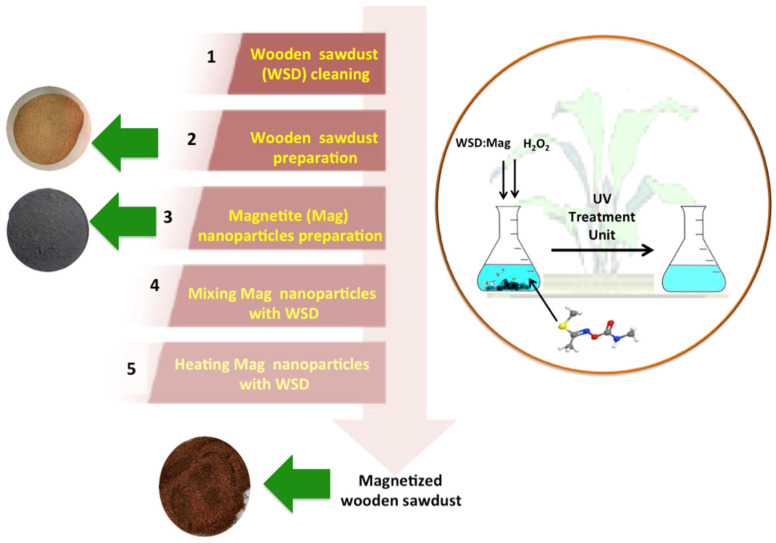
WSD:Mag preparation steps for becoming a photocatalyst.

Mag nanoparticles and treated WSD were mixed together in a proportion of 1:2 of magnetite to cellulosic WSD. Subsequently, an agate mortar was used to grind the mixture and the blend wetted with droplets of distilled water, then exposed to 5 min of heating in a microwave oven. The resultant composite was a brownish homogeneous mixture. An illustration of the WSD:Mag preparation steps and application is given in Figure 1.

### 2.2. Preparation of Nudrin Wastewater and Fenton’s Reagent Solution

Nudrin (S-methyl-N((methylcarbamoyl)oxy)thioactimidate) is an insecticide used in greenhouse farming of ornamental herbaceous plants, and was used in the current study as a model pollutant. Nudrin has high solubility in water (57.9 g/L, 20 °C). A stock aqueous solution of 1000 ppm was prepared from Nudrin and the solution was further diluted as required to attain the required concentrations. The synthetic aqueous solution of Nudrin was subjected to ultraviolet illumination, and the WSD:Mag catalyst was added at different concentrations. Hydrogen peroxide (H_2_O_2_, 30% *w*/*v*) was used to initiate the Fenton reagent reaction. The pH of the Nudrin aqueous solution was adjusted when required to the desired values by using H_2_SO_4_or NaOH (Sigma-Aldrich, St. Louis, MO, USA). All the chemicals used in treatments were used as received from the supplier without further purification or treatment.

### 2.3. Characterization of Wooden Sawdust–Magnetite Nanocomposite (WSD:Mag)

The phase structure of the synthesized WSD:Mag composite was investigated using a Phillips X’pert (MPD3040) diffractometer. The intensity of the diffracted X-rays obtained in the X-ray diffraction pattern (XRD) was recorded with a scan step of 0.02° over the range 10–70°. The morphology of the synthesized WSD:Mag nanoparticle composite was also explored with a JEM-2100 transmission electron microscope (TEM). Size distribution was investigated using ImageJ 1.48.

### 2.4. Nudrin Photo-Oxidation Experiments

From the prepared Nudrin solution, 100 mL was poured into a 250 mL glass container and pH adjusted if needed prior to the WSD:Mag being added. Hydrogen peroxide reagent was added to initiate the Fenton oxidation reaction. The aqueous solution and the reagents were then exposed to ultrasonic dispersion to verify the catalyst well dispersion in the solution. The reaction was conducted under ultraviolet (UV) illumination through mechanical stirring to precede the photocatalytic reaction. At set time intervals after the reaction, samples were withdrawn periodically for analysis. The samples were subjected to microfiltering to retain the heterogeneous catalyst prior to spectrophotometric analysis. All analysis was conducted in triplicate, and the data given are the averages of the three analyses.

### 2.5. Analysis

At a maximal wavelength of 231 nm of Nudrin pesticide, a UV-vis spectrophotometer (Unico UV-2100, Franksville, WI, USA) was used to determine the residual Nudrin concentration. The samples were withdrawn at set time intervals, and prior to measurements being taken, the nanoparticles were separated from the aqueous solution using a microfilter. When the samples’ pH was required to be adjusted, a digital pH meter (AD1030, Adwa instrument, Szeged, Hungary) was used.

### 2.6. Box–Behnken Regression Design (BBRD)

For gaining a full understanding of the role of the independent variables on the Nudrin oxidation system, measured as Nudrin removal (dependent variable), a three-level factorial design with triplicates of the central values, namely Box–Behnken factorial design, was used. The three selected parameters as the independent variables were hydrogen peroxide concentration, WSD:Mag dosing, and pH value in preliminary experiments (as seen in Table 1). Box–Behnken factorial regression design (BBRD) was used. The full factorial experimental design matrix is tabulated in Table 2. Analysis of variance (ANOVA) and graphical representation were conducted, and SAS (SAS, Institute USA) and Matlab software (7.11.0.584) were applied for both statistical and graphical analysis of the data to check the adequacy of the model. The full 15 runs of the design were carried out and the predicted design results supplemented with experimental data are shown in Table 2. The corresponding second-order polynomial equation of the model was obtained to illustrate the relationship between the Nudrin response removal and operating independent parameters. The corresponding quadratic equation is written according to the following Equation (1).

(1) $$\zeta =\beta_{o}+\sum \beta_{i}X_{i}+\sum \beta_{ii}X_{i}^{2}+\sum \beta_{ij}X_{i}X_{j}$$

where ζ is the predicted Nudrin removal rate dependent variable response (%); *i* = 1, 2, 3 and *j* = 1, 2, 3; *β_o_*, *β_i_*, *β_ii_* and *β_ij_* are the model regression coefficient parameters; and *X_i_* is the input controlling coded parameter. The natural uncoded parameters of the system design (X*_i_*) were transferred to coded variables (ε*_i_*) in accordance with Equation (1) to simplify the model calculations. Mathematical software (version 5.2) was used to locate the optimum values and then further additional experiments were carried out for model verification.

## 3. Results and Discussion

### 3.1. Structural and Morphological Characterization

#### 3.1.1. XRD Analysis

**Figure 2:**
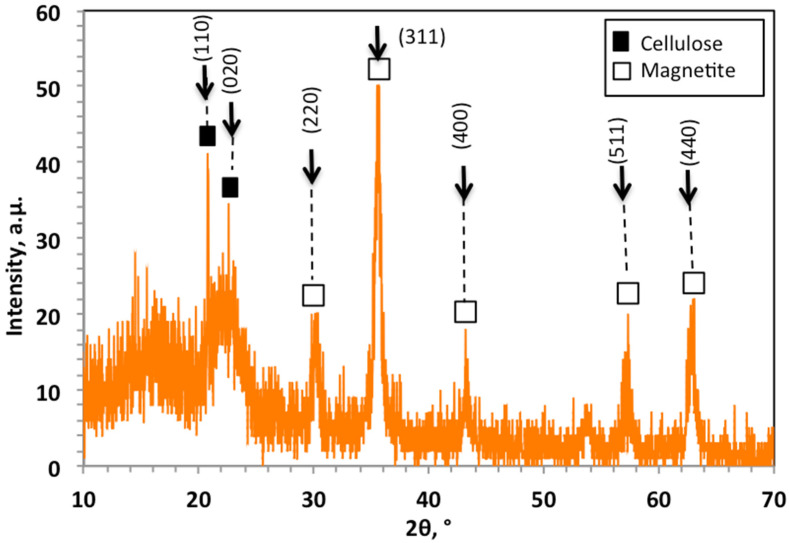
XRD patterns of WSD:Mag photocatalyst composite.

Figure 2 displays the XRD patterns of the prepared WSD:Mag nanoparticle composite in 2θ range from 10° to 70°. Cellulose and magnetite crystalline materials were defined, and the crystalline planes were identified. These crystalline phases in the XRD pattern of Figure 2 showed two sharp peaks of cellulose I at 2θ (degrees) of 22.4° and 2θ of 15.6°, which corresponded to (101) and (002) lattice planes [3,22]. Moreover, the magnetite nanoparticles displayed by diffraction peaks at 2θ (degrees) of 30.15°, 35.61°, 43.25°, 57.37°, 62.81°and 71.33° corresponded to the Miller indices (220), (311), (400), (511) and (440), respectively, which confirmed the crystal phase of Fe_3_O_4_ nanoparticles with a spinel structure [23,24].

#### 3.1.2. TEM Images

**Figure 3:**
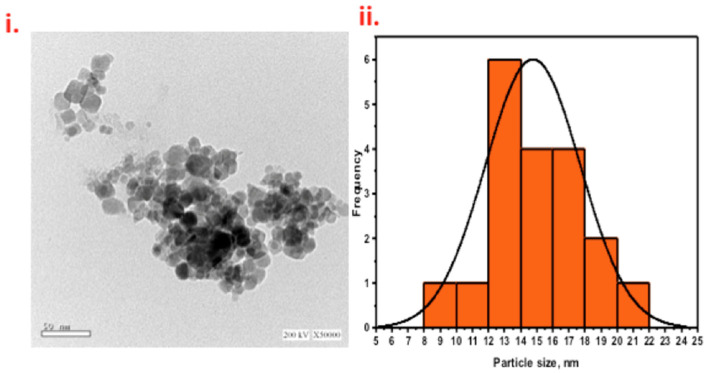
TEM images of WSD:Mag nanoparticle composite material (**i**) and particle size distribution (**ii**).

The composite material of cellulosic sawdust fibers (WSD) and magnetite (Mag) nanoparticles were investigated (Figure 3i). TEM data showed that the nonuniform sheet morphology indicated the presence of cellulosic sawdust material. The synthesized Fe_3_O_4_ nanoparticles were nearly spherical and augmented the sheet-like particles of the cellulosic sawdust. The particles of magnetite were dense and dispersed in a nonuniform coverage of the WSD surface. Figure 3ii shows the particle size distribution histogram. The recorded average particle size was 14.7 (nm), which is quite a reasonable size to offer a high surface area for the photocatalytic reaction.

### 3.2. Nudrin Photocatalytic Oxidation

#### 3.2.1. Effect of Reaction Time and Different Oxidation Systems

**Figure 4:**
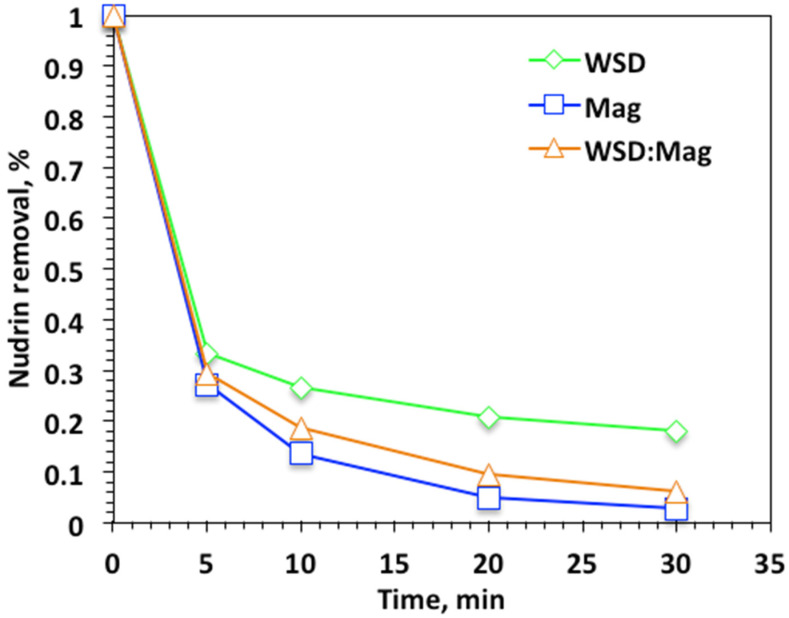
Effect of reaction time on photo-Fenton-like oxidationon different systems.

Experimental parameters influencing the reaction kinetics were explored, starting with the experimental reaction time. The influence of Fenton oxidation was examined through Nudrin removal efficiency. Initially, experiments were undertaken for reaction times ranging from 5 to 30 min to explore the reaction time for various systems—WSD/H_2_O_2_, Mag/H_2_O_2_, and WSD:Mag/H_2_O_2_—keeping the initial Nudrin concentration at 50 ppm and 40 and 400 mg/L of WSD and Mag, respectively, at a solution pH of 3.0. Data in Figure 4 reveal that the effect of reaction time on the profile of Nudrin oxidation varied once the oxidation system was altered, i.e., WSD/H_2_O_2_, Mag/H_2_O_2_, and WSD:Mag/H_2_O_2_. A higher initial oxidation drop was achieved in the first 5 min for all applied systems and subsequently steadily decreased; however, the final oxidation was obtained at 30 min of irradiance time. Overall, the magnetite-based Fenton reaction system showed higher Nudrin removal efficiency, which reached 97%. However, the WSD:Mag-based system still exhibited high removal efficiency (93%) in comparison to the WSD-based system, which showed only 82%. The rapid oxidation in the initial time period was associated with ˙OH radicals generation that might have steadily diminished with time. A reduction in hydrogen peroxide reaction occurred with time, and the creation of other less active radicals, such as hydroperoxyl radicals (HO_2_), which inhibited the oxidation rate rather than enhancing it. Therefore, the Nudrin oxidation capacity is declined as time elapsed. As the reaction proceeded, hydroxyl free radical loading declined corresponding to the decline in hydrogen peroxide [25,26]. Results of Thabet et al. [24] and Soliman et al. [8] verify such results.

Notably, when the solo WSD catalyst was applied, the Nudrin removal was also associated with the coagulation reaction. Thus, the reaction yield wasnot as high as the magnetite-based reactions. However, in the magnetite-based systems, magnetite played a significant role in enhancing the photo-oxidation reaction and producing further OH radicals in the reaction media. Although the solo magnetite system exhibited higher Nudrin oxidation efficiency (97%) than the WSD:Mag-based system (93%), the presence of WSD material with magnetite material at a ratio of 2:1, respectively, revealed the sustainability of the catalyst, showing that using waste material from sawdust to treat another aqueous waste stream satisfied the concept of circular economy.

#### 3.2.2. Initial Nudrin Concentration

**Figure 5:**
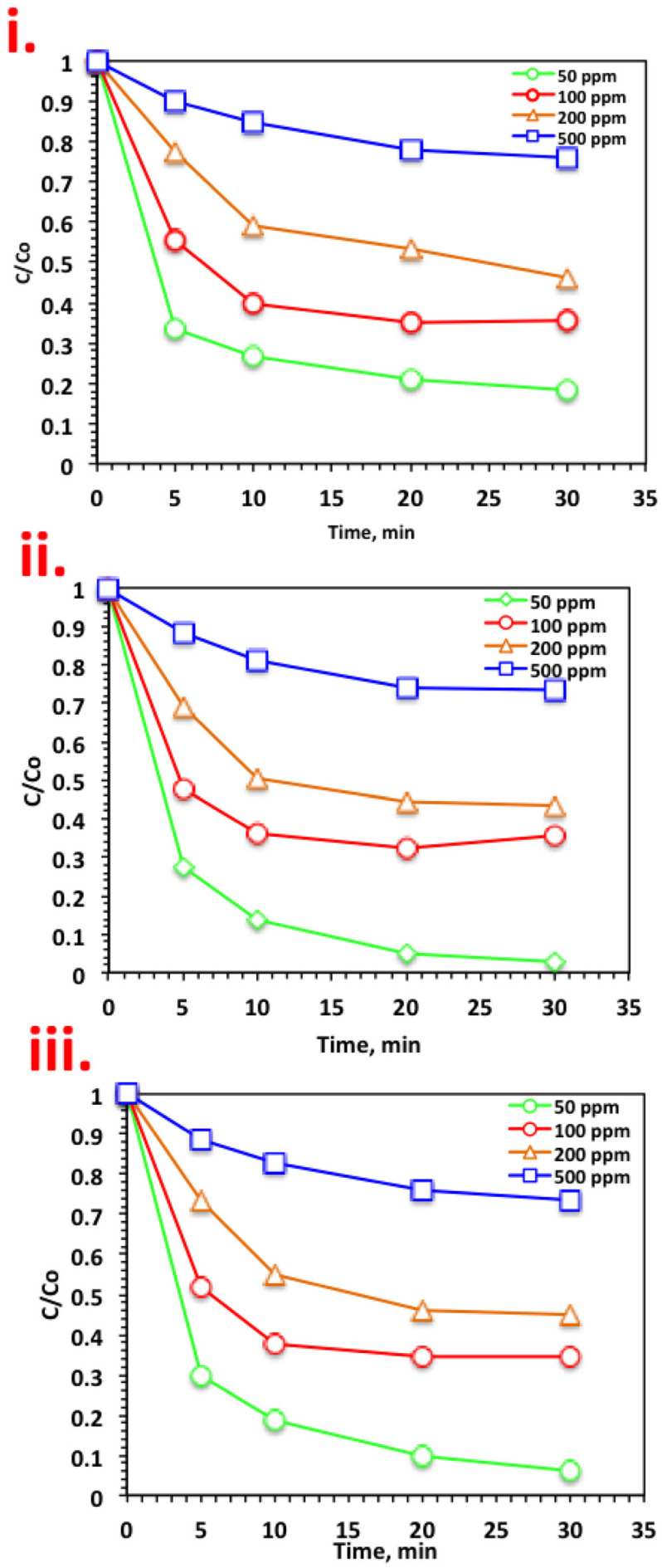
Effect of Nudrin loading on different systems: (**i**) WSD-based Fenton; (**ii**) Mag-based Fenton, and (**iii**) WSD:Mag composite-based Fenton.

For practical use and real-life applications, it was essential to check the various Nudrin loading on the oxidation system. For the different systems, namely, WSD/H_2_O_2_, Mag/H_2_O_2_, and WSD:Mag/H_2_O_2_, the initial Nudrin loading was investigated and the results are displayed in Figure 4. For the all systems in Figure 5i–iii, the same rate of rapid oxidation was recorded in the initial time period, but the oxidation rate declined with increasing Nudrin load. The removal efficiencies are 93%, 65%, 55%, and 26% using the WSD:Mag/H_2_O_2_ for 50, 100, 200, and 500 ppm of Nudrin, respectively (Figure 5iii). A similar trend was achieved for all the systems, whereas the oxidation efficiency declined from 97% to 27% with the increase in Nudrin loading from 50 to 500 ppm, respectively, for the solo magnetite-based Fenton system (Figure 5ii). The WSD/H_2_O_2_ exhibited a reduction from 81% to 23% with the Nudrin loading increase from 50 to 500 ppm, respectively (Figure 5i).

The decline in the removal rate with the increase in Nudrin loading was illustrated by the amount of catalyst (WSD, magnetite or composite) and H_2_O_2_ reagents as constants while the amount of pollutant increased. Thus, at higher Nudrin concentrations, the concentration of ˙OH radicals generated was insufficient to attain the same removal efficiency as at low Nudrin concentrations, as observed by Nitoi et al. [27], who used photo-Fenton reagent for the oxidation of lindane. Furthermore, Najjar et al. [25] recorded a decreasing of the oxidation rate with increasing initial organic load.

#### 3.2.3. Effect of Photo-Fenton-like Parameters

##### Effect of H_2_O_2_ Concentration

**Figure 6:**
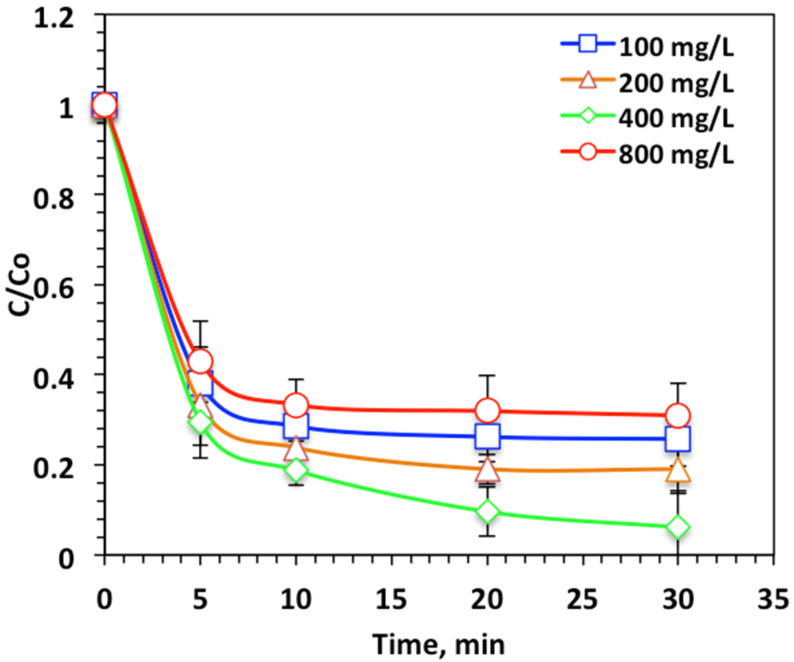
Effect of hydrogen peroxide loading on photo-Fenton-like oxidation-based WSD:Mag system.

The effect of the H_2_O_2_ loading on the Nudrin removal from pesticide-containing aqueous solution is displayed in Figure 6. As expected, a pronounced increase in the oxidation efficiency was obtained with increasing the peroxide reagent dose from 100 to 400 mg/L. The oxidation efficiency increased from 74% to 93% with hydrogen peroxide concentration increase. Although an excessive concentration of peroxide dosing reached 800 mg/L resulted in a decline in Nudrin oxidation efficiency to 69%. This enhancement in the oxidation rate with the increasing hydrogen peroxide dose is associated with the extra ˙OH radical generated due to hydrogen peroxide decomposition [7]. Conversely, in the case of excessive hydrogen peroxide dosing, the radical generated is instead the hydroperoxyl radical (HO_2_˙), which has insignificant oxidation capabilities and is weaker than the ˙OH radical. Hence, the oxidation reaction rate declined as the highly oxidizing radical (OH) yield declined [24]. The optimal operating H_2_O_2_ concentration was found to be 400 mg/L for maximum Nudrin removal for the photo-Fenton-like modified system. Previous findings confirm this result. For instance, Raut-Jadhav et al. [28], Tamimi et al. [29] and Mico et al. [30] found the oxidation rate declined with H_2_O_2_ concentrations in excess of the optimum dose for the homogeneous Fenton systems.

##### Effect of WSD:Mag Dose

**Figure 7:**
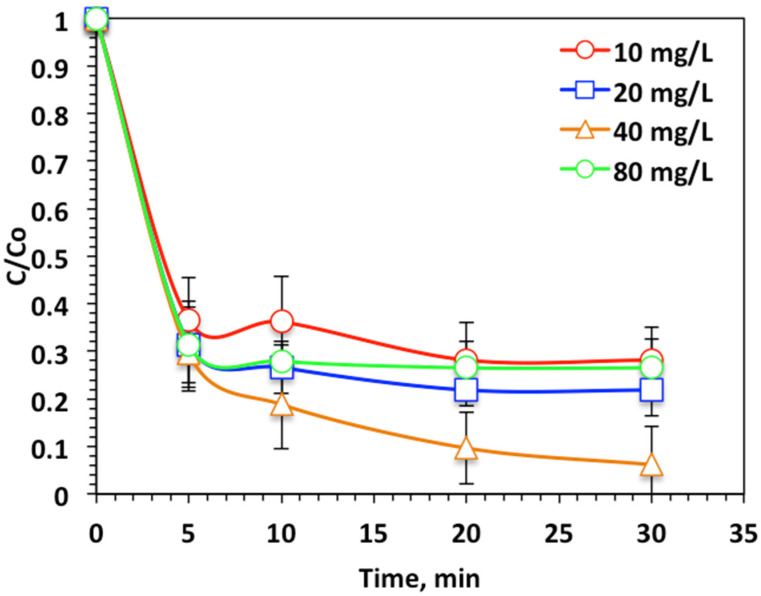
Effect of WSD:Mag loading on photo-Fenton-like oxidation-based WSD:Mag system.

To explore the impact of WSD:Mag nanoparticle composite on the photo-Fenton-like oxidation of Nudrin, experiments were undertaken to inspect the influence of composite doses on reaction kinetics. Figure 7 demonstrates its effect on the oxidation process by altering the WSD:Mag concentration over the range 10 to 80 mg/L. The data displayed in Figure 7 reveal that the oxidation tendency was enhanced from 72% to 93% when the WSD:Mag concentration increased from 10 to 40 mg/L, respectively, whereas a further increase in the WSD:Mag nanoparticle composite (80 mg/L) in the reaction medium resulted in a decline in the Nudrin reduction and oxidation tendency (73%). The WSD:Mag nanoparticle composite was critical in creating the photoactive intermediates that absorb photons in ultraviolet light and further generate highly reactive OH radicals. Fe^2+^ and Fe^3+^ are formed in the reaction medium and then react with the hydrogen peroxide reagent to produce more ˙OH radicals and iron species [7,24]. Such hydroxyl radicals are nonselective and attack the Nudrin molecules and strongly oxidize them [25]. An increase in WSD:Mag nanoparticles composite to a dose of 80 mg/L results in an upsurge in oxidation efficiency, but excessive WSD:Mag ion dosing results in a decline in the reaction rate. This could be illustrated by iron acting as an OH radical scavenger rather than a producer of them [31]. Moreover, at higher WSD:Mag concentration, a highly colored and a turbid solution was obtained, hence the ultraviolet illumination light transmission through the aqueous medium was deduced [29]. Therefore, the hydrogen peroxide photolysis to generate ˙OH radicals declined. The result was a decline in the overall oxidation reaction rate. Hence, overdosing WSD:Mag into photo-Fenton-like doses results in a decline in Nudrin oxidation [8,31].

##### Effect of pH Value

**Figure 8:**
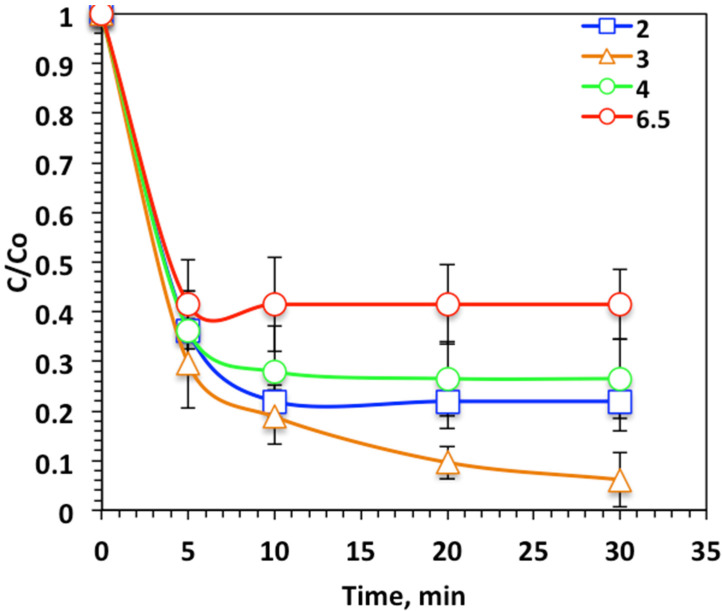
Effect of pH value on photo-Fenton-like oxidation-based WSD:Mag system.

As previously mentioned in the literature cited [32], the photo-Fenton oxidation system is sensitive to the pH of the aqueous medium. Thus, various pH values were studied to check the effect of altering the Nudrin effluent pH on oxidation efficiency. Figure 8 shows that changing the pH value form the original aqueous solution pH (6.5) to the acidic medium (4.0, 3.0, and 2.0), resulted in a decline in pH to the acidic value corresponded to an increase in Nudrin oxidation to 73% (at pH 3.0) compared to 58% (at pH 6.5), although further decline in pH to 2.0 resulted in a reduction in Nudrin removal to 78%. This result could be related to the photo-Fenton-like oxidation system working in an active manner in the acidic solution pH [33], but increasing the pH into the alkaline range results in a decline in the oxidation. This investigation is related to the pH point of zero charge—pHPZC. Commonly, the pHPZC of the WSD surface is positioned to be near the neutral value of 6.7 [23,32]. Thus, the surface of WSD is positively charged. However, elevating the solution pH into the alkaline range might weaken the attraction between the WSD and Nudrin molecules and thus the oxidation rate is declined. However, at the acidic pH, an electrostatic attraction between the positively charged WSD and the Nudrin molecule was obtained and thus the oxidation rate was enhanced. A similar study revealed such a result in treating vanadium [34]. Therefore, pH was adjusted to acidic (pH 3.0) in the further work conducted.

#### 3.2.4. Response Surface Methodology Model Analysis for System Optimization

##### Model Design Buildup

The Box–Behnken design combined with the experimental and predicted data of Nudrin photocatalytic oxidation under UV illumination using WSD:Mag nanocomposites in a Fenton reaction system are tabulated in Table 2. The Box–Behnken design was used to optimize the prompting parameters on the oxidation reaction. A quadratic second-order polynomial expression shown in Equation (2) validates the associated response function on Nudrin removal.

(2) $$\zeta \left(\%\right)=94.36-9.41\varepsilon_{1}-2.51\varepsilon_{2}-2.90\varepsilon_{3}-15.51\varepsilon_{1}^{2}-1.99\varepsilon_{1}\varepsilon_{2}-1.76\varepsilon_{1}\varepsilon_{3}\;-8.15\varepsilon_{2}^{2}+4.04\varepsilon_{2}\varepsilon_{3}-36.39\varepsilon_{3}^{2}$$

where $\varepsilon_{1}$, $\varepsilon_{2}$ and $\varepsilon_{3}$ are the coded levels of hydrogen peroxide, catalyst concentrations and pH, respectively.

##### Fitting Model and Analysis of Variance (ANOVA)

At this point, analysis of variance (ANOVA) was used investigate the suitability of the proposed model for Nudrin removal (Table 3). The correlation coefficient (*R*^2^) as well as Fisher’s F-value and probability values were used to check the quality of the fit of the polynomial model. The response variability explained by the model was accepted, since the *R*^2^ value was 93%. From this value, it can be stated that a good correlation was obtained, indicating good model fit, for which *R*^2^ of at least 0.80 is needed [35]. This was obtained from ANOVA at 93% confidence (*p* < 0.05), which corresponds to 0.02 in the proposed model.

After model suitability was confirmed, the next point was the identification of significant variables and/or interactions based on Student’s *t*-test. According to the data displayed in Table 3, the *X*_1_ (H_2_O_2_) and *X*_2_ (WASD:Mag) doses had a positive effect on the response (*p* < 0.05 for 93% confidence level). However, after a certain limit, both doses had a negative effect on the response. Subsequently, *X*_1_ (H_2_O_2_) and *X*_3_ (pH) had a stronger effect on the response.

**Figure 9:**
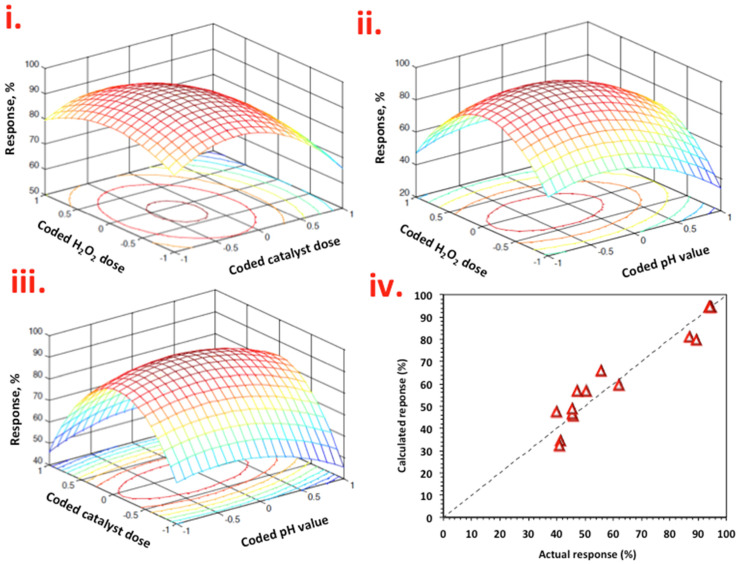
Graphical representation of Box–Behnken response surface design for Nudrin removal as a function of (**i**) 3-D surface and contour plot of coded H_2_O_2_ dose and WSD dose, (**ii**) 3-D surface and contour plot of coded H_2_O_2_ dose and pH, (**iii**) 3-D surface and contour plot of coded WSD dose and pH and (**iv**) actual and predicated responses.

MATALB software was used to produce response surface plots. In Figure 9i–iii, the response surface plots are displayed and represented as a function of *X*_1_ (H_2_O_2_) and *X*_2_ (WASD:Mag) (Figure 9i), *X*_1_ (H_2_O_2_) and *X*_3_ (pH) in Figure 9ii and *X*_2_ (WASD:Mag) and *X*_3_ (pH) in Figure 9iii.

From Figure 9i–iii, the key parameter for Nudrin oxidation was H_2_O_2_ concentration. The effect of the WASD:Mag catalyst was significant, but to a lesser extent. H_2_O_2_ and WASD:Mag concentrations affect positively the Nudrin removal and oxidation. This is due to photolysis of iron species in the WSD:Mag composite producing extra hydroxyl radicals that further reacted with more hydrogen peroxide molecules, but when H_2_O_2_ and WASD:Mag moves to higher doses, a detrimental effect on Nudrin oxidation was obtained, which can be associated with the scavenging of radicals. Thus, significant H_2_O_2_ and WASD:Mag doses are needed for photo-Fenton reactions to occur. The worst results were obtained at 50 and 500 mg/L for WAS:Mag and H_2_O_2_, respectively (the lowest level of concentrations used).

Mathematica software (V 5.2) was used to locate the optimized values from the quadratic second-order polynomial equation. The values were 385 and 38 mg/L for hydrogen peroxide and WAS:Mag catalyst, respectively, at an optimal pH value of 2.5 with a predicted response of 94%. At this point, a punctual Nudrin oxidation and removal measure was performed after attaining the optimum conditions under photo-Fenton treatment. An additional experiment was performed and the response reached 94.5%. The data obtained were compared with the predicted response, and were well correlated.

#### 3.2.5. Temperature Effects on Kinetic and Thermodynamic Parameters

**Figure 10:**
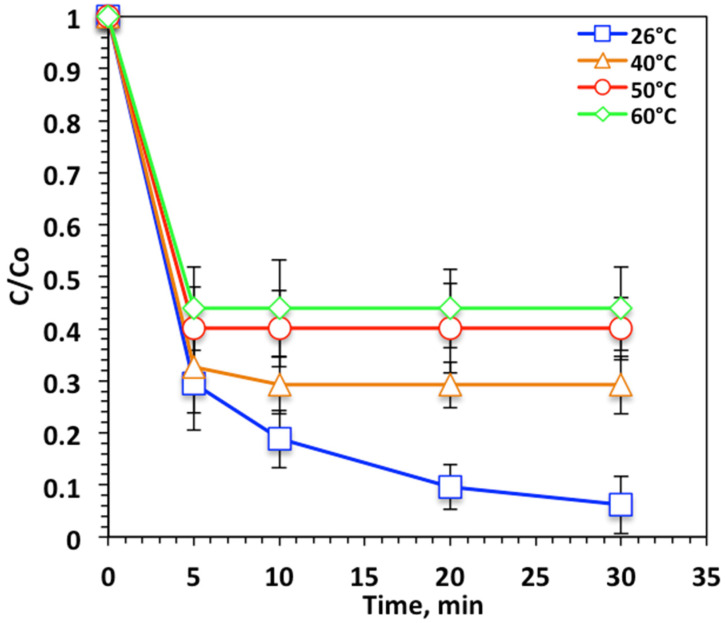
Effect of temperature on Nudrin removal by the photo-Fenton-like oxidation.

Temperature influence is categorized as an energetic parameter, since in real life, the aqueous stream could be at various temperatures, which is associated with its effect on the oxidation rate and reaction kinetics. Tests were carried out with temperatures of 26–60 °C and the results are presented in Figure 10. Figure 10 shows that the oxidation efficiency declined with increasing temperature of the medium. The elevation in temperature to 60 °C resulted in a reduction in efficiency to only oxidize 56% compared to 93% at room temperature.

Examination of Figure 10 reveals that over the temperature range 26 °C to 60 °C, a significant reduction in Nudrin oxidation removal efficiency was found. This was attributed to the H_2_O_2_ decomposes into O_2_ and H_2_O at high temperatures, which further inhibited hydroxyl radical generation, the horsepower in the Fenton oxidation system. The result was an inhibition in the overall reaction rate [36,37]. Previous studies [38,39] found temperature had a small effect on Fenton systems; however, other research confirmed that the optimum temperature for Fenton systems is 17–38 °C [35,40].

The reaction kinetics of the nanoparticle WSD:Mag system based on photo-Fenton-like reaction was studied for various contact times varying from 0 to 30 min under isothermal circumstance at the following operational temperatures: 26, 40, 50 and 60 °C. Zero-, first-, and second-order reaction kinetic models were applied to investigate the suitability of the model to examine the relationship of Nudrin removal via modified Fenton system. The linearized form of such models was applied that corresponded to zero-, first-, and second-order reactions.

By plotting the linearized form of the kinetic models for the experimental data (plots are not shown), the kinetic parameters were investigated for each model as well as the regression coefficients (*R*^2^), which were used to check the model efficiency. Table 4 contains the data for each model, showing that the reaction was best fitted by a second-order reaction model. The second-order reaction constant *k*_2_ was notably influenced by the reaction temperature, decreasing from 0.0086 to 0.0025 L/mg min with temperature increase over the examined range. Such data show that at lower temperature, the hydroxyl radical species as a result of reacting ASD:Mag composite with H_2_O_2_ increases, which further means enhancement of the reaction rate [20]. Another kinetic parameter that was significant and important was reaction half-life, *t*_1/2_, signifying as the time needed for the reactant concentration to decline to half of its initial concentration [25]. Table 4 shows that the *t*_1/2_ is a function of reaction temperature that increases with the temperature elevation.

In summary, the photo-Fenton reaction using WAS:Mag as a modified catalyst for Nudrin elimination from aqueous effluent followed a second-order reaction model over the studied temperature range. Other studies also found that Fenton treatment followed a second-order reaction [41,42,43], but Bounab et al. [44] recorded that the electro-Fenton treatment system in treating m-cresol followed a first-order kinetic model.

**Figure 11:**
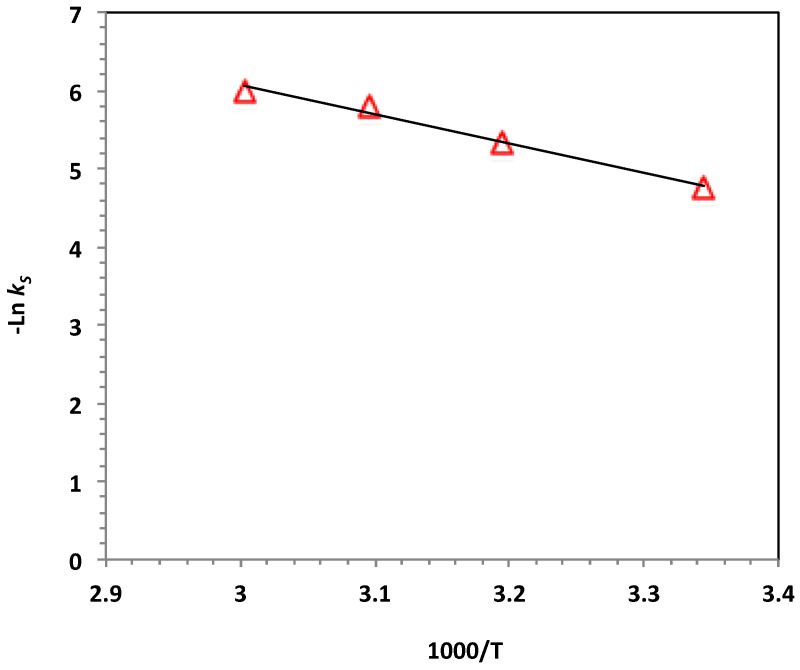
Plot of lnk2 versus 1000/T for the photo-Fenton-like reaction (solid lines represent least-squares fitting).

To investigate the photo-Fenton-like oxidation system based on WSD:Mag/H_2_O_2_, the parametric values were quantified. The activation energy (*E_a_*) of the Nudrin oxidation was explored by applying the Arrhenius formula for the kinetic rate constant, $k_{2}=Ae^{\frac{-E_{a}}{RT}}$, where *R* is the gas constant (8.314 J/mol.K), T is the temperature (in Kelvin) and A is the pre-exponential factor that is considered constant with respect to temperature. The natural log of the Arrhenius formula results in Equation (3). From Equation (2), a plot of $\ln k_{2}$ versus 1/*T* (Figure 11) presented a straight-line relationship with a slope of ($-E_{a}/R),$ which was used to estimate $E_{a}$. The activation energy of the system was 31.46 kJ/mol.

(3) $$\ln k_{2}=\ln A-\frac{E_{a}}{RT}$$

Various thermodynamic parameters, i.e., enthalpy of activation (∆H`), entropy of activation (∆S`), and free energy of activation (∆G`) were assessed employing Eyring’s equation using $E_{a}$ and $k_{2}$ according to the relation in Equation (4) [44]. Equations (5) and (6) are rearrangements of Equation (4).

(4) $$k_{2}=\frac{k_{B}T}{h}e^{\left(-\frac{\Delta G^{`}}{RT}\right)}$$

(5) $$d\left(lnk_{2}\right)/dT=E_{a}/RT^{2}$$

(6) $$lnk_{2}=\ln\left(\frac{k_{B}T}{h}\right)-\frac{\Delta H^{`}}{RT}+\frac{\Delta S^{`}}{R}$$

The activation enthalpy can be estimated from Equation (4), $\Delta H^{`}=E_{a}-RT$, and the entropy of activation, $\Delta S^{`}=\left(\Delta H^{`}-\Delta G^{`}\right)/T$, where k_B_ is Boltzmann constant and h is Planck’s constant. Accordingly, the thermodynamic data are displayed in Table 5. Positive values of activation energy ($\Delta H^{`})$ over the temperature range signify that the reaction was exothermic. Gibbs free energy of activation $\Delta G^{`}$ exhibited positive values, meaning the process was not spontaneous. This could be a result of the formation of a well-solvated structure between the Nudrin molecules and ˙OH radicals that was also maintained by a negative entropy of activation [35].

It is essential to compare the current suggested modified system with data cited previously in the literature regarding Nudrin treatment to verify the significance of the current investigation. Table 6 shows data for the modified photo-Fenton-like type reaction based on WSD/H_2_O_2_ from the current investigation in other studies. Although our value reached just 94% compared to 99% and 100% of Nudrin removal in the other cited systems in the literature, the current system is based on a cradle-to-cradle management approach. The current catalyst possesses the characteristic of magnetite, a superparamagnetic material introduces as a recoverable catalyst. Thus, the current study introduces a cost-efficient catalyst for Fenton’s reaction with atoxic end products, such as sludge formation in other systems. The current catalyst renders the sludge by-product formation from the system, which is an obstacle in the classical homogeneous Fenton system. Hence, compared to the other systems listed in Table 6, the WSD:Mag-based system is a superior environmentally benign technique for treating Nudrin.

#### 3.2.6. Catalyst Recyclability and Reuse

**Figure 12:**
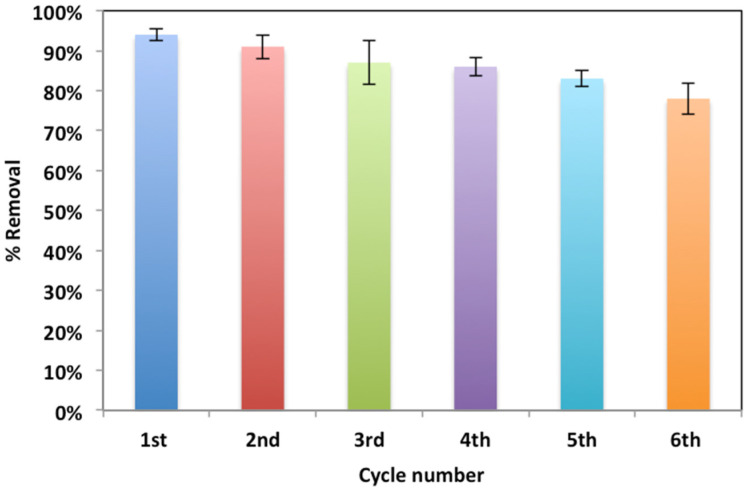
Catalyst recyclability cycles.

In order to verify the modified Fenton system sustainability concept, five treatment cycles were conducted using WSD:Mag catalyst after fresh composite use. For successive treatments, the catalyst was collected and activated after each use by filtration followed by washing with distilled water and then drying in an electric oven at 105 °C for 1 hour. For the same irradiance time, the successive use of the catalyst in the form of Fenton reaction oxidation revealed a reasonable decline in the WSD:Mag catalyst. The data displayed in Figure 12 confirm that oxidation was 78% for the sixth cycle and 94% for the first. This could be due to the WSD:Mag loaded with Nudrin molecules occupying the composite active sites and therefore hindering the oxidation rate [7]. Furthermore, the catalyst loss after each cycle was determined and found to be less than 12%. Such results confirm the sustainability of the catalyst.

## 4. Conclusions

A waste-to-cradle approach was applied and proven reliable in Nudrin wastewater treatment using wooden sawdust augmented with environmentally benign magnetite nanoparticles under photo-Fenton conditions. This oxidation system depends on several parameters. After preliminary runs, which demonstrated that pH should be maintained at 3, an empirical relationship between Nudrin oxidation removal and independent system parameters was obtained. This relationship followed a quadratic second-order polynomial equation and the optimization procedure produced high and significant correlation coefficients that verified good accordance between the model and experimental data. Values were 385 and 38 mg/L for hydrogen peroxide and WSD:Mag concentrations and pH 2.5 for the photo-Fenton treatment. Among the studied variables, the largest effect was related to hydrogen peroxide and pH. The results showed that the reaction could be carried out efficiently at room temperature. Under these conditions and with a 30 min illumination time, it was possible to oxidize 94% of Nudrin pesticide. Catalyst reusability confirmed its sustainability. It could be suggested that the system can be efficient for the real wastewater applications.

## Figures and Tables

**Table 1:** Coded levels and independent variables used in Box–Behnken design.

Experimental Variable	Symbol		Codified Levels		
			Low	Medium	High
	Natural	Coded	−1	0	1
Hydrogen peroxide (mg/L)	$X_{1}$	$\varepsilon_{1}$	300	400	500
WSD:Mag dose (mg/L)	$X_{2}$	$\varepsilon_{2}$	30	40	50
pH	$X_{3}$	$\varepsilon_{3}$	2.0	2.5	3.0

**Table 2:** Box–Behnken experimental design of WSD:Mag-based oxidation system of the three experimental variables in coded units and experimental and predicted response.

Run No.	Coded Variables			Response ( ζ, %)	
	$\varepsilon_{1}$	$\varepsilon_{2}$	$\varepsilon_{3}$	Experimental	Predicted
1	−1	−1	0	87.05	80.63
2	−1	1	0	89.48	79.59
3	1	−1	0	55.92	65.80
4	1	1	0	50.37	56.78
5	0	−1	−1	62.07	59.28
6	0	−1	1	46.06	45.38
7	0	1	−1	45.48	46.16
8	0	1	1	45.66	48.44
9	−1	0	−1	47.33	56.53
10	1	0	−1	41.29	34.19
11	−1	0	1	40.11	47.20
12	1	0	1	41.11	31.90
13	0	0	0	94.00	94.36
14	0	0	0	94.80	94.36
15	0	0	0	94.30	94.36

**Table 3:** Estimates of the model regression for Nudrin reduction under WSD:Mag-based oxidation process.

Source of Variation	Degrees of Freedom	Sum of Squares	Mean Square	F-Value	*p*-Value
Regression	9	6485.354	720.5949	6.381047	0.027552
Linear	3	826.39042	826.39042	7.317892	1.061819
Quadratic	3	916.4847	916.4847	8.115699	1.514403
Cross Product	3	5202.28272	5202.28272	46.067507	0.682254
Error	5	564.6369	112.9274		
Total	14	7049.991			
*R*^2^ = 93%					

**Table 4:** Comparison of different kinetic models for WSD:Mag-based oxidation process.

T (°C)	Zero-Order Reaction *			First-Order Reaction **			Second-Order Reaction ***		
	*k*_o_	t_1/2_ (min)	R^2^	*k*_1_	t_1/2_ (min)	R^2^	k_2_, (L.mg^−1^ min ^−1^)	t_1/2_ (min)	R^2^
	(min ^−1^)			(min ^−1)^					
26	1.96	12.83	0.65	0.11	6.35	0.89	0.0086	2.31	0.99
40	1.45	17.35	0.5	0.05	13.50	0.53	0.0048	4.14	0.9
50	1.21	20.86	0.46	0.04	18.93	0.46	0.003	6.62	0.8
60	1.13	22.28	0.46	0.03	21.00	0.46	0.0025	7.94	0.8

* $\left(\frac{dc}{dt}\right)=-k_{o}$, **$\;\left(\frac{dc}{dt}\right)=-k_{1}C$, *** $\left(\frac{dc}{dt}\right)=-k_{2\;}C^{2}$ where C is the concentration of wastewater.

**Table 5:** Thermodynamic properties for Nudrin removal by for WSD:Mag-based oxidation process.

Thermodynamic Parameters	Temperature (°C)			
	26	40	50	60
∆G` (kJ/mol)	85.06	90.67	94.92	98.45
∆H` (kJ/mol)	28.97	28.85	28.77	28.69
∆S`(J/molK)	−187.57	−197.51	−204.78	−209.48
Ea (kJ/mol)	31.46			

**Table 6:** Comparison of different treatment methodologies with WSD:Mag-based oxidation systems for Nudrin remediation *.

Treatment Process	Effluent Characteristics/Operating Parameters	Performance Efficiency, %	Ref.
Ultraviolet oxidation-based WSD:Mag system	50 ppm-_Nudrin_; WSD:Mag 38 mg/L; H_2_O_2_ 386 mg/L; pH 2.5; T 26 °C	94%	Present work
Ultraviolet oxidation-based AS:M system	50 ppm-_Nudrin_; AS:M 50 mg/L; H_2_O_2_ 130 mg/L; pH 6; T 30 °C	99%	[45]
Ultraviolet oxidation—iron nanomaterial-based system	50 ppm-_Nudrin_; Fe^2+^ 40 mg/L^;^ H_2_O_2_ 50 mg/L; pH 3; T 35 °C	90%	[46]
Solar oxidation—iron nanomaterial-based system	50 ppm-_Nudrin_; Fe^2+^ 44 mg/L; H_2_O_2_ 52 mg/L; pH 3	96.5%	[46]
Ultraviolet oxidation—copper nanomaterial-based system	50 ppm-_Nudrin_;n-CuO80 mg/L; H_2_O_2_ 400 mg/L; pH 6.5	99%	[47]
Sonication oxidation—iron nanomaterial-based system	25 ppm-_Nudrin_; Fe^2+^/H_2_O_2_ 1:30 mM; pH 2.5	100%	[28]
Ultraviolet oxidation—titanium nanomaterial-based system	50 ppm-_Nudrin_; TiO_2_ 20 mg/LH_2_O_2_ 400 mg/L; pH 6.5; T 25 °C	100%	[48]
Microwave oxidation—cupper nanomaterial-based system	200 ppm-_Nudrin_; Cu-catalyst 3 g/L;H_2_O_2_ 5000 mg/L	91%	[49]
Ultraviolet oxidation—iron/zeolite-based system	16.22 ppm-_Nudrin_; Fe-ZSM-5 zeolite 5 g/L; pH 3.7; T 25 °C	100%	[50]
Ultraviolet oxidation- Zinc based system	16.22 ppm-_Nudrin_;ZnO 2000 mg/L; pH 5.6; T 25 °C	80%	[51]
Ultraviolet oxidation- Ozone based system	20 ppm-_Nudrin_; Ozone 3600 mg/L; pH 3.24–7.2; T 25 °C	100%	[52]
Ultraviolet oxidation- Sand/iron based system	100 ppm-_Nudrin_;sand/iron 45 mg/L; H_2_O_2_ 103 mg/L; pH 2.8	96%	[9]

* WSD:Mag: wooden sawdust: magnetite; AS:M: alum sludge: magnetite.

## Data Availability

Data available upon Request.
